# Supplementary material for: FERN – a Java framework for stochastic simulation and evaluation of reaction networks
Source: BMC Bioinformatics. 2008 Aug 29;9:356. doi: 10.1186/1471-2105-9-356 (PMC2553347; doi:10.1186/1471-2105-9-356)
Supplement: Additional file 1 — FERN distribution, Version 1.3. This archive contains the FERN source code and binaries as well as documentation and example models in FernML and SBML. [file 1471-2105-9-356-S1.zip › fern/doc/javadoc/fern/network/Network.html]

Network


---


|  |  |  |
| --- | --- | --- |
| **Overview** \| **Package** \| **Class** \| **Use** \| **Tree** \| **Deprecated** \| **Index** \| **Help** | |  |
| **PREV CLASS**   **NEXT CLASS** | **FRAMES**    **NO FRAMES**     **All Classes** |  |
| SUMMARY: NESTED \| FIELD \| CONSTR \| METHOD | DETAIL: FIELD \| CONSTR \| METHOD |  |


---


## fern.network Interface Network

**All Known Implementing Classes:**: AbstractNetworkImpl, AutocatalyticNetwork, CatalysedNetwork, CellDesignerNetworkWrapper, CytoscapeNetworkWrapper, ExtractSubNetwork, FernMLNetwork, ModifierNetwork, ReversibleNetwork, SBMLNetwork

---

``` public interface Network ```

The central interface of the whole framework. Each `Simulator` takes a `Network`
for simulation, as well as each analysis algorithm takes one. Additionally `Network`s
can be modified or saved as FernML-Files.

Basically only the networks structure is stored here, other things like management of
the species populations (amounts), propensities or annotations is sourced out to separate
classes. The advantage is that you can reuse existing `AmountManager`s,... when
you are implementing your own network (reader, wrapper, evolver,...) when you think, one
of the existing ones is suitable.

**Author:**
:   Florian Erhard

**See Also:**: `AnalysisBase`, `FernMLNetwork.FernMLNetwork(Network)`, `FernMLNetwork.saveToFile(java.io.File)`, `ModifierNetwork`

---

| **Method Summary** | |
| --- | --- |
| `AmountManager` | `getAmountManager()`             Gets the `AmountManager` for this network. |
| `AnnotationManager` | `getAnnotationManager()`             Gets the `AnnotationManager` for this network. |
| `long` | `getInitialAmount(int species)`             Gets the initial amount of the specified molecule species. |
| `String` | `getName()`             Gets an identifier of the network. |
| `int` | `getNumReactions()`             Gets the number of reaction within the network. |
| `int` | `getNumSpecies()`             Gets the number of species within the network. |
| `int[]` | `getProducts(int reaction)`             Gets the products of the specified reaction. |
| `PropensityCalculator` | `getPropensityCalculator()`             Gets the `PropensityCalculator` for this network. |
| `int[]` | `getReactants(int reaction)`             Gets the reactants of the specified reaction. |
| `String` | `getReactionName(int index)`             Gets a string representation of the reaction. |
| `int` | `getSpeciesByName(String name)`             Gets the species index by name. |
| `String` | `getSpeciesName(int index)`             Gets the species name by index. |
| `void` | `setInitialAmount(int species, long value)`             Sets the initial amount of the specified molecule species. |

**Method Detail**

### getAmountManager

```
AmountManager getAmountManager()
```

:   Gets the `AmountManager` for this network.

    :   **Returns:**: the `AmountManager`

---


### getPropensityCalculator

```
PropensityCalculator getPropensityCalculator()
```

:   Gets the `PropensityCalculator` for this network.

    :   **Returns:**: the `PropensityCalculator`

---


### getAnnotationManager

```
AnnotationManager getAnnotationManager()
```

:   Gets the `AnnotationManager` for this network.

    :   **Returns:**: the `AnnotationManager`

---


### getNumSpecies

```
int getNumSpecies()
```

:   Gets the number of species within the network.

    :   **Returns:**: number of species

---


### getNumReactions

```
int getNumReactions()
```

:   Gets the number of reaction within the network.

    :   **Returns:**: number of reactions

---


### getReactants

```
int[] getReactants(int reaction)
```

:   Gets the reactants of the specified reaction.

    :   **Parameters:**: `reaction` - index of the reaction **Returns:**: indices of the reactants

---


### getProducts

```
int[] getProducts(int reaction)
```

:   Gets the products of the specified reaction.

    :   **Parameters:**: `reaction` - index of the reaction **Returns:**: indices of the products

---


### getSpeciesByName

```
int getSpeciesByName(String name)
```

:   Gets the species index by name. If the argument is no valid species, -1 is returned.

    :   **Parameters:**: `name` - name of the species **Returns:**: index of the species

---


### getSpeciesName

```
String getSpeciesName(int index)
```

:   Gets the species name by index.

    :   **Parameters:**: `index` - index of the species **Returns:**: name of the species

---


### getReactionName

```
String getReactionName(int index)
```

:   Gets a string representation of the reaction.

    :   **Parameters:**: `index` - reaction index **Returns:**: string representation

---


### getName

```
String getName()
```

:   Gets an identifier of the network.

    :   **Returns:**: identifier of the network

---


### getInitialAmount

```
long getInitialAmount(int species)
```

:   Gets the initial amount of the specified molecule species.

    :   **Parameters:**: `species` - index of the species **Returns:**: initial amount of the species

---


### setInitialAmount

```
void setInitialAmount(int species,
                      long value)
```

:   Sets the initial amount of the specified molecule species.

    :   **Parameters:**: `species` - index of the species: `value` - initial amount of the species


---


|  |  |  |
| --- | --- | --- |
| **Overview** \| **Package** \| **Class** \| **Use** \| **Tree** \| **Deprecated** \| **Index** \| **Help** | |  |
| **PREV CLASS**   **NEXT CLASS** | **FRAMES**    **NO FRAMES**     **All Classes** |  |
| SUMMARY: NESTED \| FIELD \| CONSTR \| METHOD | DETAIL: FIELD \| CONSTR \| METHOD |  |


---
